# Gain-of-Function Mutations in the Phospholipid Flippase MprF Confer Specific Daptomycin Resistance

**DOI:** 10.1128/mBio.01659-18

**Published:** 2018-12-18

**Authors:** Christoph M. Ernst, Christoph J. Slavetinsky, Sebastian Kuhn, Janna N. Hauser, Mulugeta Nega, Nagendra N. Mishra, Cordula Gekeler, Arnold S. Bayer, Andreas Peschel

**Affiliations:** a Interfaculty Institute of Microbiology and Infection Medicine, Infection Biology, University of Tübingen, Tübingen, Germany; b German Centre for Infection Research (DZIF), Partner Site Tübingen, Tübingen, Germany; c Los Angeles Biomedical Research Institute at Harbor-UCLA Medical Center, Torrance, California, USA; d David Geffen School of Medicine at UCLA, Los Angeles, California, USA; e Department of General Pediatrics, Oncology/Hematology, University Children’s Hospital Tübingen, Tübingen, Germany; MedImmune

**Keywords:** daptomycin, MRSA, MprF, *Staphylococcus aureus*, antibiotic resistance, flippase

## Abstract

Daptomycin, a calcium-dependent lipopeptide antibiotic whose full mode of action is still not entirely understood, has become a standard-of-care agent for treating methicillin-resistant Staphylococcus aureus (MRSA) infections. Daptomycin-resistant (DAP-R) S. aureus mutants emerge during therapy, featuring isolates which in most cases possess point mutations in the *mprF* gene. MprF is a bifunctional bacterial resistance protein that synthesizes the positively charged lipid lysyl-phosphatidylglycerol (LysPG) and translocates it subsequently from the inner membrane leaflet to the outer membrane leaflet. This process leads to increased positive S. aureus surface charge and reduces susceptibility to cationic antimicrobial peptides and cationic antibiotics. We characterized the most commonly reported MprF mutations in DAP-R S. aureus strains in a defined genetic background and found that only certain mutations, including the frequently reported T345A single nucleotide polymorphism (SNP), can reproducibly cause daptomycin resistance. Surprisingly, T345A did not alter LysPG synthesis, LysPG translocation, or the S. aureus cell surface charge. MprF-mediated DAP-R relied on a functional flippase domain and was restricted to daptomycin and a related cyclic lipopeptide antibiotic, friulimicin B, suggesting that the mutations modulate specific interactions with these two antibiotics. Notably, the T345A mutation led to weakened intramolecular domain interactions of MprF, suggesting that daptomycin and friulimicin resistance-conferring mutations may alter the substrate range of the MprF flippase to directly translocate these lipopeptide antibiotics or other membrane components with crucial roles in the activity of these antimicrobials. Our study points to a new mechanism used by S. aureus to resist calcium-dependent lipopeptide antibiotics and increases our understanding of the bacterial phospholipid flippase MprF.

## Abstract (precis)

Ever since daptomycin was introduced to the clinic, daptomycin-resistant isolates have been reported. In most cases, the resistant isolates harbor point mutations in MprF, which produces and flips the positively charged phospholipid LysPG. This has led to the assumption that the resistance mechanism relies on the overproduction of LysPG, given that increased LysPG production may lead to increased electrostatic repulsion of positively charged antimicrobial compounds, including daptomycin. Here we show that the resistance mechanism is highly specific and relies on a different process that involves a functional MprF flippase, suggesting that the resistance-conferring mutations may enable the flippase to accommodate daptomycin or an unknown component that is crucial for its activity. Our report provides a new perspective on the mechanism of resistance to a major antibiotic.

## INTRODUCTION

The opportunistic pathogens Staphylococcus aureus, Enterococcus faecalis, and Enterococcus faecium are responsible for a large percentage of invasive infections, particularly in hospitalized and immunocompromised patients (1). These often life-threatening infections are complicated by the high prevalence of methicillin-resistant S. aureus (MRSA) and vancomycin-resistant enterococci (VRE) (1, 2), resulting in the frequent use of the lipopeptide antibiotic daptomycin, which has remained effective against drug-resistant isolates. Yet, an increasing number of reports on mutations emerging during daptomycin therapy, leading to increased daptomycin minimal inhibitory concentrations (MICs), have been reported (3 – 5). This phenomenon has raised concerns about the future of daptomycin therapy for such pathogens and has led to demands for in-depth investigations on the resistance mechanisms as well as countermeasures (6, 7).

Daptomycin is the first approved drug of a new class of calcium-dependent lipopeptide antibiotics, whose entire mode of action is still not fully understood (6, 8). It interacts with phosphatidylglycerol (PG) and interferes with bacterial fluid membrane microdomains, which leads to inhibition of cell wall synthesis (9). However, it is unclear if its activity requires a specific docking molecule in the membrane (9, 10). How point mutations in different S. aureus proteins contribute to or cause DAP-R remains equally elusive (6). Several proteins involved in the synthesis, regulation, or maintenance of cell surface molecules have been reported to be mutated during prolonged exposure to daptomycin (6). The characterization of such mutants has led, in part, to conflicting findings of potentially altered bacterial cell surface charge, lipid composition, or cell wall thickness (3, 4, 11 – 15). In S. aureus, distinct mutations in the phospholipid (PL) synthase and flippase, MprF, have repeatedly been found to affect daptomycin susceptibility (6) and to be among the first to emerge during exposure of S. aureus to serial passage in increasing sublethal daptomycin concentrations (16). MprF links lysine to negatively charged PG (17) and translocates the resulting positively charged lysyl-PG (LysPG) to the outer leaflet of the cytoplasmic membrane (CM) (18), resulting in electrostatic repulsion of cationic antimicrobial peptides (CAMPs), including human defensins and bacterial lantibiotics (17, 18). Daptomycin resembles CAMPs following its binding to calcium ions, an event absolutely required for its microbiologic activity (19). Chromosomal deletion of MprF leads to CAMP and daptomycin hypersusceptibility, while an intact MprF protein confers a basic level of DAP-R, which is usually low enough to enable effective therapy with daptomycin (18).

MprF is the first example of a bacterial phospholipid flippase. Recent studies have revealed important details about its membrane topology and domain organization (20). It is still unclear which lipid molecules can be translocated by MprF, although its substrate range has been found to include the zwitterionic lipid alanyl-PG (AlaPG) in addition to LysPG (21). Of note, DAP-R-conferring point mutations in MprF are often found in distinct regions of the protein and do not seem to affect conserved amino acid positions (6, 18, 20).

In this study, we characterized the most frequently reported DAP-R-associated MprF point mutations in a defined genetic background, in order to exclude potentially contributing activities of additional mutations and accessory elements. We found that only some of the widely reported mutations associated with DAP-R can reproducibly cause DAP-R and that they do not affect LysPG synthesis and translocation or any other process affecting the S. aureus cell surface charge. MprF-mediated DAP-R led to cross-resistance only to the structurally related cyclic lipopeptide friulimicin B, which has a different target than daptomycin (10, 22), indicating that the resistance mechanism is based on specific interactions with the drug rather than the target molecule. We found that DAP-R relied on a functional flippase domain and was associated with reduced intramolecular domain interactions of MprF, suggesting that alteration of the protein structure modulates the substrate range of the flippase to accommodate daptomycin or another membrane-embedded substrate that is crucial for daptomycin activity.

## RESULTS

### Distinct point mutations at the junction of MprF synthase and flippase lead to DAP-R.

**FIG 1:**
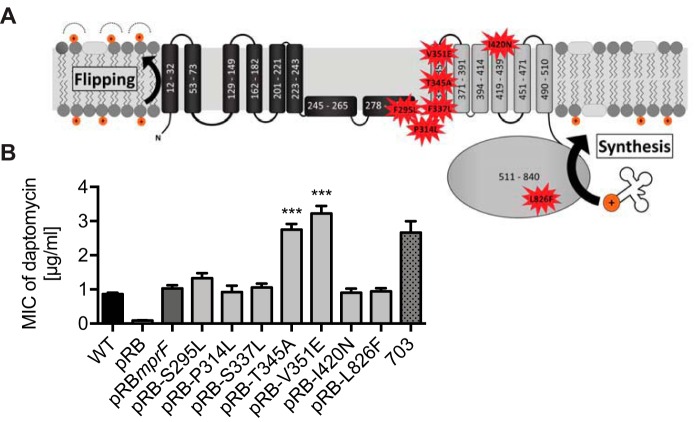
Specific mutations at the junction of the flippase domain and synthase domain of MprF confer daptomycin resistance. (A) Topology of most frequent DAP-R-associated point mutations in MprF (Table S1). The MprF synthase and flippase domains are shown in gray and black, respectively. (B) Impact of daptomycin resistance-associated point mutations expressed in the S. aureus *ΔmprF* mutant on daptomycin susceptibility. The recently characterized clinical daptomycin-resistant isolate, strain 703 (4), served as a control and reference for clinically relevant daptomycin MICs. Values that are significantly different from the values determined for the S. aureus *ΔmprF* mutant expressing wild-type MprF (pRB*mprF*) are indicated (***, *P* < 0.0001). The means plus standard errors of the means (SEM) of results from at least five independent experiments are shown. WT, wild type.

The most frequently identified MprF mutations associated with DAP-R are located at the junction of the flippase domain and synthase domain or in the synthase domain of the protein (Fig. 1A; see also Table S1 in the supplemental material). These strains often contain additional point mutations in other chromosomal loci, such as *yycFG* (*walKR*), *rpoB*, *rpoC*, *vraS*, and *dltA* (6, 7), raising the issue of whether the documented MprF mutations are in fact sufficient for mediating the DAP-R phenotype. In order to elucidate the contribution of individual mutations to DAP-R in a defined genetic background, the most frequently identified mutations were introduced into *mprF* harbored on a plasmid, which was then transferred to the S. aureus 113 (SA113) *mprF* mutant. Two mutations at the junction between the flippase domain and the synthase domain (T345A and V351E) led to significantly increased, clinically relevant DAP-R (MIC of 3 µg/ml) compared to the parental MprF sequence (MIC of 1 µg/ml) (Fig. 1B). In contrast, other mutations in this region of the protein (S295L, P314L, and S337L) and two mutations in the synthase domain (I420N and L826F) did not alter daptomycin susceptibility, suggesting that these mutations contribute to DAP-R only in combination with additional mutations. Thus, specific mutations at the junction between the synthase domain and the flippase domain of MprF can reproducibly confer DAP-R in S. aureus.

10.1128/mBio.01659-18.2 TABLE S1 Frequency of reported point mutations in MprF. The frequency of the reported mutations is shown. A distinction is made between the frequency of mutations reported from *in vitro* passaging studies and the frequency of mutations reported from studies with clinical isolates. Download Table S1, DOCX file, 0.1 MB. Copyright © 2018 Ernst et al. 2018 Ernst et al. This content is distributed under the terms of the Creative Commons Attribution 4.0 International license.

### DAP-R-conferring point mutations in MprF do not alter the cellular LysPG level or membrane leaflet distribution.

**FIG 2:**
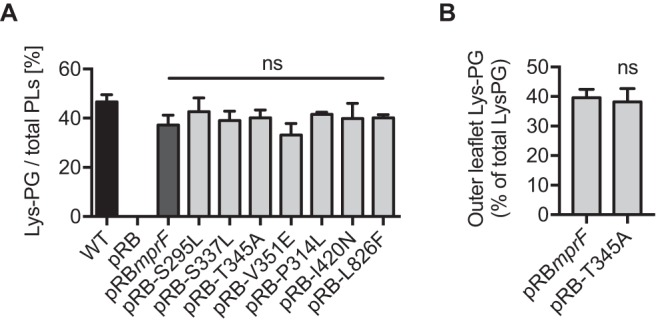
Signature DAP-R mutations in MprF do not alter known functions of MprF. (A) Percentages of LysPG production in relation to total phospholipid (PL) content. (B) Percentages of LysPG located in the outer leaflet of the membrane. Values that are not significantly different from the values determined for the S. aureus *ΔmprF* mutant expressing wild-type MprF (pRB*mprF*) are indicated (ns). The means plus SEM of results from three independent experiments are shown.

Basal levels of CAMP resistance mediated by MprF depend on the protein’s capacity to synthesize LysPG and translocate a substantial amount of this lipid to the outer membrane leaflet, where it repulses harmful cationic proteins by electrostatic interaction (17, 18, 23, 24). In order to elucidate if the DAP-R-associated point mutations at the junction between the synthase domain and the flippase domain (Fig. 1B) might lead to increased activity of one of the two protein domains, the levels of LysPG production and distribution between the inner and outer membrane leaflets of S. aureus with native versus mutated *mprF* were compared. LysPG contents were determined by thin-layer chromatography (TLC) and staining of LysPG with the phosphate group-specific dye molybdenum blue (20, 21). None of the S. aureus strains expressing a mutated *mprF* gene exhibited altered LysPG production (Fig. 2A), indicating that these point mutations confer DAP-R via a mechanism other than increasing LysPG synthesis. Of note, the expression of the cloned *mprF* variants was controlled by the constitutive Bacillus subtilis promoter *vegII*, which may explain why they displayed slightly reduced LysPG production compared to the wild type. The localization of LysPG in the cytoplasmic membrane was determined by incubating intact S. aureus cells expressing MprF with wild-type sequence or with the T345A mutation with the fluorescent dye fluorescamine, which reacts with the free amino group of LysPG at the outer membrane leaflet but cannot access the inner leaflet. Thin-layer chromatography and quantification of fluorescamine-labeled versus nonlabeled LysPG allowed inner-leaflet and outer-leaflet LysPG to be distinguished (25, 26). Experiments performed with wild-type MprF and T345A-MprF led to the same percentage of LysPG in the outer membrane (ca. 40%) (Fig. 2B), indicating that DAP-R is not associated with an increased capacity of MprF to translocate LysPG. Thus, the signature mutations in MprF leading to DAP-R do not seem to alter either of the two documented activities of MprF.

### The DAP-R-conferring MprF point mutation T345A does not alter the S. aureus surface charge.

**FIG 3:**
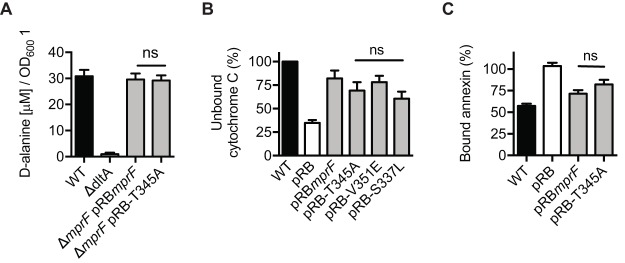
The DAP-R-conferring point mutations T345A and V351E do not affect cell surface charge. (A) Quantification of teichoic acid d-alanylation. The SA113 *dltA* deletion mutant served as a negative control (43). (B) Percentages of repulsed cytochrome *c* normalized to the wild type. The *mprF* deletion mutant was used as a negative control. (C) Percentages of bound annexin V normalized to the *ΔmprF* mutant harboring the empty plasmid (pRB). The *mprF* deletion mutant was used as a negative control. Values that are not significantly different from the values determined for the S. aureus *ΔmprF* mutant expressing wild-type MprF (pRBmprF) are indicated (ns). The means plus SEM of results from three independent experiments are shown.

Daptomycin is thought to integrate into the bacterial cytoplasmic membrane upon binding of calcium ions in a manner similar to that seen with many typical CAMPs (19). Most bacteria achieve protection against a broad range of CAMPs by introduction of positive charges that modify the bacterial surface charge and diminish the affinity for CAMPs, thereby allowing bacteria to tolerate substantial CAMP concentrations (27). Aside from the modification of membrane phospholipids with lysine or other amino acids, the neutralization of negatively charged teichoic acids (TAs) with d-alanine is a particularly widespread CAMP repulsion mechanism found in several bacterial divisions (27, 28). d-Alanylation of teichoic acids is mediated by the DltABCD system, which is composed of four proteins responsible for activation, transfer, and linkage of cytosolic d-alanine residues onto the backbone of teichoic acids (29). In order to determine if mutated, DAP-R-conferring MprF affects the Dlt system, we quantified the teichoic acid d-alanylation of S. aureus expressing wild-type MprF compared to T345A-MprF (Fig. 3A). While a *dltA* knockout mutant serving as a negative control showed the complete absence of d-alanylation, we did not observe a difference between strains expressing wild-type MprF and strains expressing T345A-MprF. In order to analyze if the daptomycin resistance-causing point mutations in MprF could somehow affect the overall S. aureus surface charge in a LysPG or wall teichoic acid (WTA) alanylation-independent manner, the capacities of S. aureus expressing MprF with wild-type sequence or a T345A mutation to bind the cationic protein cytochrome *c* or calcium-bound annexin V was compared. These model proteins were proven to allow a sensitive assessment of changes in the surface charge of S. aureus in several previous studies (17, 18, 21, 30). The lack of *mprF* had a profound impact on the capacity of S. aureus to bind cytochrome *c* or annexin V (Fig. 3B), demonstrating the suitability of the assays. However, the T345A mutation did not alter the binding behavior of annexin V significantly (Fig. 3C), and both DAP-R-conferring mutations (T345A and V351E) did not alter the binding of cytochrome *c* (Fig. 3B). Thus, the DAP-R-conferring point mutations in MprF do not lead to a general alteration of the cell surface charge.

10.1128/mBio.01659-18.1 FIG S1 Calcium supplementation does not confer T345A-MprF mediated resistance to calcium-independent antibiotics. Download FIG S1, EPS file, 0.1 MB. Copyright © 2018 Ernst et al. 2018 Ernst et al. This content is distributed under the terms of the Creative Commons Attribution 4.0 International license.

### MprF point mutation T345A causes cross-resistance only to daptomycin and the related lipopeptide antibiotic friulimicin B.

**FIG 4:**
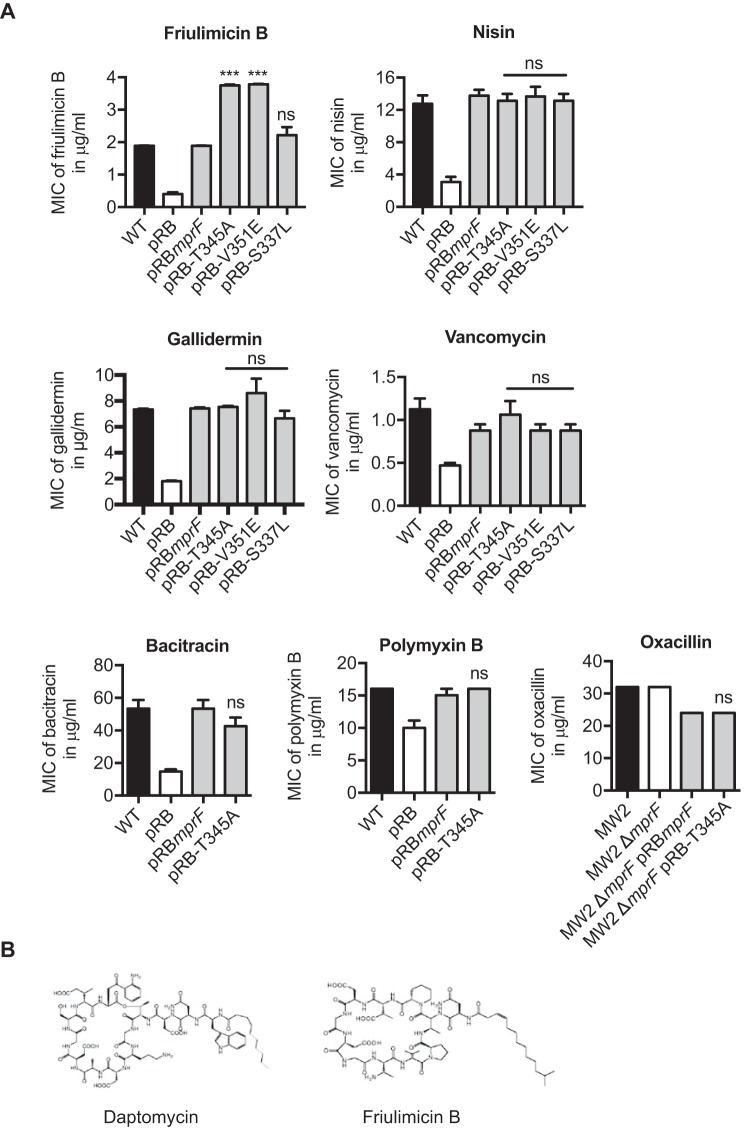
MprF-mediated daptomycin resistance leads to cross-resistance to friulimicin B. (A) MICs of antibiotics as indicated. The *mprF* deletion mutant served as a negative control. (B) Structures of friulimicin B and daptomycin (22). Values that are significantly different from the values determined for the S. aureus *ΔmprF* mutant expressing wild-type MprF (pRB*mprF*) are indicated (***, *P* < 0.0001). Values that are not significantly different from the values determined for the S. aureus *ΔmprF* mutant expressing wild-type MprF (pRB*mprF*) are indicated (ns). The means plus SEM of results from three independent experiments are shown.

The DAP-R-conferring point mutations in MprF do not seem to be based on a canonical CAMP resistance strategy, which raises the issue of how specific the resistance mechanism may be. A variety of cationic, membrane-active antibiotics from different classes and with different modes of action, including the calcium-dependent lipopeptides daptomycin and friulimicin B, the calcium-independent lipopeptide polymyxin B, the nonlipidated peptide bacitracin, the glycopeptide vancomycin, and the lantibiotics nisin and gallidermin (31, 32), were analyzed for their capacity to inhibit growth of S. aureus expressing MprF with wild-type sequence or a T345A mutation. The ability of these compounds to inhibit S. aureus growth was reduced in the presence of a functional MprF protein (Fig. 4A), which indicates that surface charge alterations have a strong impact on the capacity of these agents to inhibit S. aureus. In contrast, the neutral antibiotic oxacillin was not affected by MprF. However, the T345A and V351E mutations in MprF exclusively led to cross-resistance to friulimicin B, which is the closest relative of daptomycin among the tested antibiotics (Fig. 4) but has a different target (10). Of note, the addition of calcium to calcium-independent antibiotics did not lead to differences in the levels of inhibition of S. aureus expressing wild-type versus T345A-MprF by any of these compounds (Fig. S1). Thus, *mprF*-mediated DAP-R does not lead to broad-spectrum cross-resistance to cationic antibiotics and antimicrobial peptides but is restricted to compounds with a specific, daptomycin-related structure. Moreover, the different targets of daptomycin and friulimicin B suggest that the resistance mechanism does not involve the target of daptomycin.

### T345A-mediated DAP-R depends on the presence of a functional MprF flippase domain.

**FIG 5:**
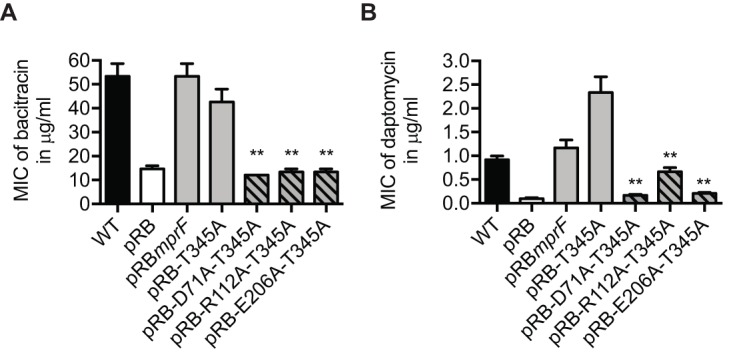
The functionality of the MprF flippase is required for MprF-mediated DAP-R. (A) MIC of bacitracin as indicator of flippase activity. (B) MIC of daptomycin. The means plus SEM of results from three independent experiments are shown. Values that are significantly different from the values determined for the S. aureus *ΔmprF* mutant expressing T345A-MprF (pRB-T345A) are indicated (**, *P* < 0.01).

The T345A point mutation does not alter the LysPG flippase activity of MprF, but it may enable the flippase to translocate other substrate molecules in addition to LysPG. We have previously identified conserved amino acids in the flippase domains of MprF proteins and have shown that they are essential for flippase activity (20). The ability of T345A to increase the MIC of daptomycin in the presence of the flippase-inactivating mutations D71A, R112A, and E206A was investigated. All the resulting strains were hypersusceptible to the CAMP bacitracin compared to strains expressing a functional MprF (Fig. 5A), which confirms that LysPG could not be translocated from the inner layer to the outer layer of the cell membrane in these strains. T345A was not able to increase the daptomycin MIC in combination with mutations D71A and R206A and led to an only slightly increased MIC with mutation R112A (Fig. 5B), indicating that the functionality of the MprF flippase is crucial for the capacity of T345A to confer DAP-R.

### The T345A point mutation reduces intramolecular interactions of MprF domains.

**FIG 6:**
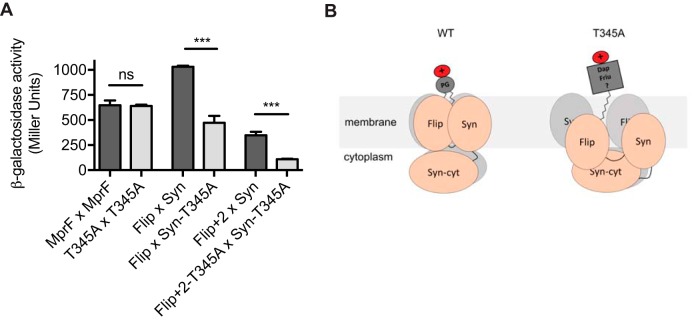
The DAP-R-conferring point mutation T345A reduces intramolecular interactions of MprF domains. The T345A mutation is located in the hydrophobic part of the synthase domain, which was previously shown to specifically interact with the flippase domain (20). β-Galactosidase activity of E. coli cells expressing full-length MprF (MprF), the flippase domain encompassing amino acids 1 to 320 (Flip), the extended flippase domain encompassing amino acids 1 to 393 (Flip + 2), and the synthase domain encompassing amino acids 328 to 840 (Syn) and T345A variants. The extended flippase domain consists of two additional transmembrane segments (TMS) of the synthase domain, which were previously shown to be required for full flippase activity (20). β-Galactosidase activity is displayed as Miller units. Values that are significantly different from those determined for the T345A variants are indicated (***, *P* < 0.0001). Values that are not significantly different from those determined for the T345A variants are indicated (ns). The means plus SEM of results from three independent experiments are shown. (B) Proposed model for MprF-mediated daptomycin resistance. MprF forms oligomers with distinct intradomain interactions (20), resulting in the formation of a translocation channel, which enables the flipping of bacterial phospholipids (LysPG and AlaPG) (21). Daptomycin resistance-conferring SNPs (e.g., T345A) reduce intradomain interactions, enabling the channel to accommodate daptomycin and friulimicin or a membrane-embedded molecule that is crucial for the activity of the two structurally related antibiotics. Flip, flippase domain; Syn, synthase domain; Syn-cyt, cytosolic part of the synthase domain; Dap, daptomycin; Friu, friulimicin B; ?, potential other membrane-embedded molecule that is crucial for daptomycin and friulimicin B activity; WT, wild type.

The point mutations in MprF leading to DAP-R do not occur at conserved amino acid positions, but they involve a variety of sites at the junction of the LysPG synthase and flippase domains (Fig. 1B). The various domains of MprF have been found to undergo several complex intramolecular interactions (20), which may be altered by the T345A point mutation. In order to test this hypothesis, the impact of T345A on the capacities of full-length MprF or of the synthase and flippase domains to interact were compared in the bacterial two-hybrid system, which has been proven to be suitable for elucidating intramolecular MprF interactions (20). Full-length MprF proteins with native sequence and those with the T345A point mutation showed similar capacities to interact (Fig. 6A). However, the flippase domain and an extended version of the flippase domain, which was previously shown to be required for full flippase activity (20), interacted with the synthase domain much less efficiently when the T345A mutation was present. Thus, the T345A mutations in MprF (leading to specific resistance to structurally related lipopeptide antibiotics) are associated with reduced intramolecular interactions. Such mutations do not seem to affect the efficiency of the flippase functionality but might instead extend the range of molecules that the flippase is able to translocate (Fig. 6B).

## DISCUSSION

Point mutations leading to resistance to antibiotics are a common phenomenon occurring during therapy with almost any antimicrobial compound (33). Resistance levels conferred by such mutations often lead to only moderately increased MICs; however, these can be sufficient to compromise the efficacy of antibiotic therapies. The mechanisms of antibiotic resistance are diverse, ranging from modified target molecules to decreased uptake or gain of function of enzymes that inactivate the antimicrobial compound (33). Elucidation of how the signature DAP-R-associated point mutations compromise the antibiotic’s activity has remained elusive (6). Since such mutations often occur within MprF, a protein which is known to electrostatically repulse CAMPs by synthesizing and translocating cationic LysPG (24), it is tempting to speculate that an increase of LysPG levels in the outer leaflet of the cytoplasmic membrane may be the major consequence of these DAP-R-conferring point mutations. By introducing the most frequently identified *mprF* mutations among clinically derived DAP-R strains into an S. aureus strain with a defined genetic background, we found, surprisingly, that many of them were not able to cause DAP-R. Yet it is possible that such mutations contribute to DAP-R in a more complex manner involving, for instance, additional changes in multiple genetic loci. Notably, our study results indicate that DAP-R-conferring point mutations at the junction of the flippase and synthase domains of MprF such as T345A and V351E do not alter the level or translocation of LysPG. Other research groups have reported the association of altered LysPG production and/or translocation with these reported *mprF* point mutations (3, 4, 14, 34). However, since most of those studies analyzed strains that had been under *in vitro* or *in vivo* selection pressure, it is possible that they harbored additional point mutations that either preexisted in the parental isolates or were acquired during daptomycin exposure that could have influenced MprF activity and DAP-R, for example, by additional modifications of the cell envelope or by other, less obvious modifications (see Table S2 in the supplemental material) (35).

10.1128/mBio.01659-18.3 TABLE S2 Phenotypes observed in DAP-R isolates. The point mutations and the strain background (clinical or generated *in vitro*) are indicated, as well as the observed phenotypes. Download Table S2, DOCX file, 0.1 MB. Copyright © 2018 Ernst et al. 2018 Ernst et al. This content is distributed under the terms of the Creative Commons Attribution 4.0 International license.

The T345A point mutation was selected for more-detailed analyses and was found to not alter LysPG production or translocation, d-alanylation of teichoic acids, or the overall cell surface charge. T345A conferred resistance to only two structurally related lipopeptide antibiotics, namely, daptomycin and friulimicin B, whereas the activity of other lipopeptide or peptide antibiotics was not affected. Since daptomycin and friulimicin B do not share the same target (10), the resistance mechanism appears to be based on specific interactions of MprF with the structurally related lipopeptide antibiotics rather than with a target molecule. Moreover, T345A could confer resistance only when the flippase domain was functional, suggesting that flippase functionality may have been extended (rather than compromised), leading to DAP-R. We have previously shown that the MprF flippase is capable of flipping two different phospholipids species, Ala-PG and LysPG (21), which indicated that the flippase has relaxed substrate specificity for similar substrates. As proposed for other phospholipid flippases (36) and as suggested by our recent structural investigation of MprF (20), the membrane-integrated domains of MprF likely associate to form a channel in order to accommodate phospholipid substrate molecules and to facilitate their translocation. Thus, the affinity of MprF domains for each other may determine the substrate specificity of the channel and, in the case of reduced domain interactions, may extend the substrate specificity of the flippase to accommodate and translocate either daptomycin and friulimicin B or another membrane-embedded molecule whose orientation in the membrane is crucial for the activity of these antibiotics (Fig. 6B). A change in MprF flippase specificity would be in agreement with all of our findings; however, this is particularly difficult to demonstrate directly because it would likely affect the orientation of substrate molecules in the membrane rather than their presence or absence. Moreover, as shown previously for other phospholipid transporters (36), the translocation process is probably very fast and may be reversible. Indeed, all our attempts to demonstrate that T345A-mutated MprF affects the membrane integration or orientation of daptomycin led to inconclusive results. Thus, future highly sophisticated and time-consuming biophysical technology performed with *in vitro*-reconstituted MprF-containing membrane vesicles will likely be necessary to study MprF-mediated altered daptomycin translocation dynamics in the membrane.

Our finding that T345A does not alter the LysPG synthase and flippase activity of MprF was unexpected and points to a novel resistance mechanism against daptomycin, which warrants further in-depth investigation. MprF is the first bacterial phospholipid flippase to have been described, but its mode of action remains only superficially understood. Our study reveals critical details of its role in a novel resistance mechanism with important implications for basic bacterial membrane-associated processes and for the development of inhibitors which may block DAP-R to maintain the efficacy of this important therapeutic compound.

## MATERIALS AND METHODS

### Bacterial strains and mutagenesis of *mprF*.

The common laboratory strain, methicillin-susceptible S. aureus SA113 (ATCC 35556) and its *mprF* knockout derivative SA113*ΔmprF* have been described recently (17). Point mutations in *mprF* were introduced by site-directed mutagenesis in Escherichia coli using E. coli/S. aureus shuttle vector pRB474 bearing *mprF* via the use of a QuikChange kit (Stratagene, La Jolla, CA, USA) (see Table S3 in the supplemental material), as described recently (20). Mutated derivatives of *mprF* were cloned in pRB474*mprF* and transferred into strain SA113*ΔmprF*. Expression of the pRB474*mprF* variants was mediated by the use of constitutive Bacillus subtilis promoter *vegII*. Plasmids were maintained with 10 μg/ml chloramphenicol in all studies, with the exception of the MIC assays. Plasmids used in this study are given in Table S3. Primers used in this study are given in Table S4.

10.1128/mBio.01659-18.4 TABLE S3 Plasmids used in this study. Download Table S3, DOCX file, 0.02 MB. Copyright © 2018 Ernst et al. 2018 Ernst et al. This content is distributed under the terms of the Creative Commons Attribution 4.0 International license.

10.1128/mBio.01659-18.5 TABLE S4 Primers used in this study. Download Table S4, DOCX file, 0.02 MB. Copyright © 2018 Ernst et al. 2018 Ernst et al. This content is distributed under the terms of the Creative Commons Attribution 4.0 International license.

### Prediction of MprF structure.

The transmembrane topology of MprF was predicted with the TOPCONS program (http://topcons.cbr.su.se/) combined with our latest experimental results (20).

### Determination of susceptibility to antimicrobial agents.

The MICs of daptomycin, bacitracin, polymyxin B, vancomycin, and oxacillin were determined with MIC test strips from Liofilchem according to the manufacturer’s advice. The MICs of friulimicin B, nisin, and gallidermin were determined by broth microdilution in Mueller-Hinton broth (MHB) in a 24-well plate under shaking conditions. Friulimicin B and daptomycin MICs were determined in the presence of 50 mg/liter CaCl_2_. Other MICs were determined in the presence of 50 mg/liter CaCl_2_ when indicated.

### Isolation and quantification of polar lipids.

Phospholipids were isolated and quantified as described recently (20, 21). Bacterial overnight cultures grown in MHB to an optical density at 600 nm (OD_600_) of 0.05 were incubated in 100 ml fresh MHB until the exponential-growth phase (OD_600_ of 0.5 to 1) was reached. After adjusting S. aureus strains to equal optical densities, the Bligh-Dyer method (37) was used to extract lipids with a chloroform-methanol-sodium acetate buffer (20 mM, pH 4.6) mixture (1:1:1 [vol/vol/vol]). Isolated lipids were vacuum dried, resuspended in chloroform-methanol (2:1 [vol/vol), and spotted onto silica gel 60 F254 high-performance thin-layer chromatography (HPTLC) plates (Merck, Darmstadt, Germany) with a Linomat 5 sample application unit (Camag, Berlin, Germany). Polar lipids were separated in an ADC 2 developing chamber (Camag, Berlin, Germany) with a chloroform-methanol-water (65:25:4 [vol/vol/vol]) running solvent. Phospholipids were detected by staining of phosphate groups with molybdenum blue, and the LysPG content was determined in relation to the total phospholipid content by densitometry analysis performed with ImageJ (http://rsbweb.nih.gov/ij/docs/guide/index.html) as described recently (20, 21).

### Translocation of LysPG.

The distribution of LysPG in the inner leaflet and outer leaflet of the membrane was determined as described recently (26). Briefly, S. aureus overnight cultures were diluted 1:100 and grown for 12 h in brain heart infusion (BHI) medium. Cells were harvested and washed several times, and the cell pellet was incubated with the membrane-impermeative, amino-reactive dye fluorescamine (0.52 M) to specifically label outer-leaflet LysPG. The reaction was stopped after 30 s, and after several washing steps, the phospholipids were extracted and separated in two dimensions via thin-layer chromatography. Fluorescamine-labeled outer-leaflet LysPG was identified with a UV lamp, while unlabeled inner-leaflet LysPG was identified with amino-group reactive ninhydrin. Both lipid species were extracted from the TLC plates and digested with perchloric acid for 3 h in order to liberate and quantify the phosphate content with a colorimetric agent and to quantify the phospholipid content spectrophotometrically at a wavelength of 660 nm.

### Quantification of d-alanine from teichoic acids.

Bacteria were grown to early stationary phase in basal medium (BM) complemented with 0.36% glucose for 6 h and washed twice with ammonium acetate buffer (20 mM, pH 4.8, 4°C) as described recently (38). A 1-ml volume of a suspension with an OD_600_ of 30 was incubated with NaOH (0.1 M; final volume of 100 μl) for 1 h of shaking at 37°C to hydrolyze the d-alanine esters. HCl (100 μl) served as a stopping reagent, and cell debris was removed by centrifugation and sterile filtration. The d-alanine content of the teichoic acid polymers was assayed by high-performance liquid chromatography (HPLC) upon precolumn derivatization of the amino acid by the use of ortho-phthalaldehyde (OPA). The sample and reagent (OPA diluted 1:10 in 1 M sodium borate buffer, pH 10.7) were drawn into the autosampler injection needle (Agilent 1200 HPLC system; Waldbronn, Germany) and shaken for 90 s before injection. The amino acid derivatives were separated on a reversed-phase column (Grom-Sil OPA-1; Alltech-Grom GmbH, Rottenburg-Hailfingen, Germany) (150 mm by 4.6 mm, 3-µm pore size) at a flow rate of 1.1 ml/min using a linear-gradient elution from 0% to 60% buffer B for 15 min and were detected at 340 nm. Buffer A was 25 mM phosphate buffer (pH 7.2) containing 0.75% tetrahydrofuran (THF), while buffer B was composed of 35% MeOH–15% acetonitrile (ACN)–25 mM phosphate buffer. A minimum of three independent runs were performed. Peak areas were quantified based on a d-alanine standard curve.

### Repulsion of cationic cytochrome *c*.

Differences in the bacterial capacity to repulse cationic proteins were determined by comparing the levels of binding of the red-colored cationic protein cytochrome *c* as described previously (18, 39). Exponential-phase bacteria were harvested and washed twice with sodium acetate buffer (20 mM, pH 4.6), and the bacterial cell suspension was adjusted to an OD_600_ of 3. Aliquots of 1.5 ml were pelleted, resuspended in 750 μl cytochrome *c* solution (Sigma; 0.25 mg/ml in sodium acetate buffer), and incubated at 37°C with shaking for 15 min. Suspensions were pelleted, the resulting supernatant was diluted 1:5 with sodium acetate buffer, and absorbance was measured at 410 nm.

### Binding of annexin V to negatively charged phospholipids.

To validate the experimentation of the phospholipids, particularly for the assay examining translocation of LysPG, we performed the annexin V-Ca^++^ assay, which measures the levels of binding to phosphatidyl serine present on the outer layer of cell membrane (“flipped”) (40). This assay has been very commonly used in eukaryotic systems to unravel apoptotic reactions, because of the ability of annexin V-Ca^++^ to bind to and demonstrate the translocation of phosphatidylserine in the outer layer of the CM. We utilized this method as an indirect measure of the relative levels of outer CM-flipped, positively charged LysPG (the higher the level of positively charged LysPG that is flipped to the outer CM, the lower the level of negatively charged PL species that are available for annexin V-Ca^++^ binding) (40 – 42). Briefly, S. aureus *cells* were grown overnight in BHI broth. Postcentrifugation, the cell pellet was washed twice and resuspended in binding buffer to adjust the OD_600_ to 0.5 (∼10^8^ CFU/ml). A 5-μl volume of allophycocyanin (APC) annexin V was added to the cells, and the cells were subjected to gentle vortex mixing and incubated at room temperature for 15 min in the dark (30). The cells were then quantified by flow cytometry for analysis of surface-bound fluorophore (30) (excitation and emission wavelengths of 650 nm and 660 nm, respectively; 10,000 events acquired). Data are represented in relative fluorescent units.

### MprF domain interactions.

MprF-domain interactions were analyzed with a bacterial two-hybrid kit (BACTH system kit; Euromedex), as described recently (20). Briefly, E. coli BTH101 was transformed with *mprF* variants (Table S1) and fused to adenylate cyclase fragments T25 and T18 of Bordetella pertussis, and protein interactions resulting in cyclic AMP (cAMP) production and subsequent expression of the *lac* and *mal* operons in E. coli were quantified by determining β-galactosidase activity in triplicate (20).
