# Supplementary material for: Gain-of-Function Mutations in the Phospholipid Flippase MprF Confer Specific Daptomycin Resistance
Source: mBio. 2018 Dec 18;9(6):e01659-18. doi: 10.1128/mBio.01659-18 (PMC6299216; doi:10.1128/mBio.01659-18)
Supplement: TABLE S1 [file mbo006184218st1.docx]

**Table S1 – Frequency of reported point mutations in MprF.** The frequency of the reported mutations is shown. A distinction is made between the frequency of mutations reported from *in vitro* passaging studies, and the frequency of mutations reported from studies with clinical isolates.

| MprF point mutation | *In vitro* isolates | Clinical isolates | Reference |
| --- | --- | --- | --- |
| E44V | 1 | 0 | (1) |
| G61V | 0 | 1 | (2) |
| L291I | 1 | 0 | (3) |
| S295L | 2 | 11 | (1, 2, 4-18) |
| A302V | 1 | 0 | (5) |
| P314L | 3 | 6 | (3, 5, 6, 12, 17-21) |
| S337L | 3 | 9 | (2, 4, 5, 12, 17-24) |
| L338S | 1 | 0 | (24) |
| L341S | 3 | 0 | (8, 11, 12, 17-19, 25) |
| T345A/I/K | 4 | 13 | (2, 6, 9, 12, 14, 17, 18, 26-31) |
| M347R | 1 | 1 | (5, 12, 18) |
| V351E | 1 | 1 | (12, 18, 32) |
| H376Y | 1 | 0 | (5) |
| I420N/S/T | 2 | 3 | (1, 2, 8, 11, 33) |
| W424C | 1 | 0 | (5) |
| T472K | 0 | 1 | (12, 18) |
| I506M | 1 | 0 | (1) |
| E692Q | 0 | 1 | (20) |
| L776S | 0 | 1 | (17) |
| L826F/I | 2 | 28 | (2, 5, 6, 8, 10-12, 17-20, 25, 28, 34) |

1. **Kosowska-Shick K, Clark C, Pankuch GA, McGhee P, Dewasse B, Beachel L, Appelbaum PC.** 2009. Activity of telavancin against staphylococci and enterococci determined by MIC and resistance selection studies. Antimicrob Agents Chemother **53:**4217-4224.

2. **Peleg AY, Miyakis S, Ward DV, Earl AM, Rubio A, Cameron DR, Pillai S, Moellering RC, Jr., Eliopoulos GM.** 2012. Whole genome characterization of the mechanisms of daptomycin resistance in clinical and laboratory derived isolates of Staphylococcus aureus. PLoS One **7:**e28316.

3. **Mishra NN, Yang SJ, Chen L, Muller C, Saleh-Mghir A, Kuhn S, Peschel A, Yeaman MR, Nast CC, Kreiswirth BN, Cremieux AC, Bayer AS.** 2013. Emergence of daptomycin resistance in daptomycin-naive rabbits with methicillin-resistant Staphylococcus aureus prosthetic joint infection is associated with resistance to host defense cationic peptides and mprF polymorphisms. PLoS One **8:**e71151.

4. **Quinn B, Hussain S, Malik M, Drlica K, Zhao X.** 2007. Daptomycin inoculum effects and mutant prevention concentration with Staphylococcus aureus. J Antimicrob Chemother **60:**1380-1383.

5. **Berti AD, Baines SL, Howden BP, Sakoulas G, Nizet V, Proctor RA, Rose WE.** 2015. Heterogeneity of genetic pathways toward daptomycin nonsusceptibility in Staphylococcus aureus determined by adjunctive antibiotics. Antimicrob Agents Chemother **59:**2799-2806.

6. **Friedman L, Alder JD, Silverman JA.** 2006. Genetic changes that correlate with reduced susceptibility to daptomycin in Staphylococcus aureus. Antimicrob Agents Chemother **50:**2137-2145.

7. **Jones T, Yeaman MR, Sakoulas G, Yang SJ, Proctor RA, Sahl HG, Schrenzel J, Xiong YQ, Bayer AS.** 2008. Failures in clinical treatment of Staphylococcus aureus Infection with daptomycin are associated with alterations in surface charge, membrane phospholipid asymmetry, and drug binding. Antimicrob Agents Chemother **52:**269-278.

8. **Mishra NN, McKinnell J, Yeaman MR, Rubio A, Nast CC, Chen L, Kreiswirth BN, Bayer AS.** 2011. In vitro cross-resistance to daptomycin and host defense cationic antimicrobial peptides in clinical methicillin-resistant Staphylococcus aureus isolates. Antimicrob Agents Chemother **55:**4012-4018.

9. **Mishra NN, Bayer AS, Weidenmaier C, Grau T, Wanner S, Stefani S, Cafiso V, Bertuccio T, Yeaman MR, Nast CC, Yang SJ.** 2014. Phenotypic and genotypic characterization of daptomycin-resistant methicillin-resistant Staphylococcus aureus strains: relative roles of mprF and dlt operons. PLoS One **9:**e107426.

10. **Bertsche U, Yang SJ, Kuehner D, Wanner S, Mishra NN, Roth T, Nega M, Schneider A, Mayer C, Grau T, Bayer AS, Weidenmaier C.** 2013. Increased cell wall teichoic acid production and D-alanylation are common phenotypes among daptomycin-resistant methicillin-resistant Staphylococcus aureus (MRSA) clinical isolates. PLoS One **8:**e67398.

11. **Mishra NN, Bayer AS.** 2013. Correlation of cell membrane lipid profiles with daptomycin resistance in methicillin-resistant Staphylococcus aureus. Antimicrob Agents Chemother **57:**1082-1085.

12. **Bayer AS, Mishra NN, Chen L, Kreiswirth BN, Rubio A, Yang SJ.** 2015. Frequency and Distribution of Single-Nucleotide Polymorphisms within mprF in Methicillin-Resistant Staphylococcus aureus Clinical Isolates and Their Role in Cross-Resistance to Daptomycin and Host Defense Antimicrobial Peptides. Antimicrob Agents Chemother **59:**4930-4937.

13. **Fischer A, Yang SJ, Bayer AS, Vaezzadeh AR, Herzig S, Stenz L, Girard M, Sakoulas G, Scherl A, Yeaman MR, Proctor RA, Schrenzel J, Francois P.** 2011. Daptomycin resistance mechanisms in clinically derived Staphylococcus aureus strains assessed by a combined transcriptomics and proteomics approach. J Antimicrob Chemother **66:**1696-1711.

14. **Yang SJ, Mishra NN, Rubio A, Bayer AS.** 2013. Causal role of single nucleotide polymorphisms within the mprF gene of Staphylococcus aureus in daptomycin resistance. Antimicrob Agents Chemother **57:**5658-5664.

15. **Yang SJ, Kreiswirth BN, Sakoulas G, Yeaman MR, Xiong YQ, Sawa A, Bayer AS.** 2009. Enhanced expression of dltABCD is associated with the development of daptomycin nonsusceptibility in a clinical endocarditis isolate of Staphylococcus aureus. J Infect Dis **200:**1916-1920.

16. **Chen CJ, Huang YC, Chiu CH.** 2015. Multiple pathways of cross-resistance to glycopeptides and daptomycin in persistent MRSA bacteraemia. J Antimicrob Chemother **70:**2965-2972.

17. **Rubio A, Moore J, Varoglu M, Conrad M, Chu M, Shaw W, Silverman JA.** 2012. LC-MS/MS characterization of phospholipid content in daptomycin-susceptible and -resistant isolates of Staphylococcus aureus with mutations in mprF. Mol Membr Biol **29:**1-8.

18. **Bayer AS, Mishra NN, Cheung AL, Rubio A, Yang SJ.** 2016. Dysregulation of mprF and dltABCD expression among daptomycin-non-susceptible MRSA clinical isolates. J Antimicrob Chemother **71:**2100-2104.

19. **Mehta S, Cuirolo AX, Plata KB, Riosa S, Silverman JA, Rubio A, Rosato RR, Rosato AE.** 2012. VraSR two-component regulatory system contributes to mprF-mediated decreased susceptibility to daptomycin in in vivo-selected clinical strains of methicillin-resistant Staphylococcus aureus. Antimicrob Agents Chemother **56:**92-102.

20. **Steed ME, Hall AD, Salimnia H, Kaatz GW, Kaye KS, Rybak MJ.** 2013. Evaluation of Daptomycin Non-Susceptible Staphylococcus aureus for Stability, Population Profiles, mprF Mutations, and Daptomycin Activity. Infect Dis Ther **2:**187-200.

21. **Kang KM, Mishra NN, Park KT, Lee GY, Park YH, Bayer AS, Yang SJ.** 2017. Phenotypic and genotypic correlates of daptomycin-resistant methicillin-susceptible Staphylococcus aureus clinical isolates. J Microbiol **55:**153-159.

22. **Boyle-Vavra S, Jones M, Gourley BL, Holmes M, Ruf R, Balsam AR, Boulware DR, Kline S, Jawahir S, Devries A, Peterson SN, Daum RS.** 2011. Comparative genome sequencing of an isogenic pair of USA800 clinical methicillin-resistant Staphylococcus aureus isolates obtained before and after daptomycin treatment failure. Antimicrob Agents Chemother **55:**2018-2025.

23. **Cameron DR, Jiang JH, Abbott IJ, Spelman DW, Peleg AY.** 2015. Draft Genome Sequences of Clinical Daptomycin-Nonsusceptible Methicillin-Resistant Staphylococcus aureus Strain APS211 and Its Daptomycin-Susceptible Progenitor APS210. Genome Announc **3**.

24. **Patel D, Husain M, Vidaillac C, Steed ME, Rybak MJ, Seo SM, Kaatz GW.** 2011. Mechanisms of in-vitro-selected daptomycin-non-susceptibility in Staphylococcus aureus. Int J Antimicrob Agents **38:**442-446.

25. **Bayer AS, Mishra NN, Sakoulas G, Nonejuie P, Nast CC, Pogliano J, Chen KT, Ellison SN, Yeaman MR, Yang SJ.** 2014. Heterogeneity of mprF sequences in methicillin-resistant Staphylococcus aureus clinical isolates: role in cross-resistance between daptomycin and host defense antimicrobial peptides. Antimicrob Agents Chemother **58:**7462-7467.

26. **Yang SJ, Nast CC, Mishra NN, Yeaman MR, Fey PD, Bayer AS.** 2010. Cell wall thickening is not a universal accompaniment of the daptomycin nonsusceptibility phenotype in Staphylococcus aureus: evidence for multiple resistance mechanisms. Antimicrob Agents Chemother **54:**3079-3085.

27. **Murthy MH, Olson ME, Wickert RW, Fey PD, Jalali Z.** 2008. Daptomycin non-susceptible meticillin-resistant Staphylococcus aureus USA 300 isolate. J Med Microbiol **57:**1036-1038.

28. **Rubio A, Conrad M, Haselbeck RJ, G CK, Brown-Driver V, Finn J, Silverman JA.** 2011. Regulation of mprF by antisense RNA restores daptomycin susceptibility to daptomycin-resistant isolates of Staphylococcus aureus. Antimicrob Agents Chemother **55:**364-367.

29. **Capone A, Cafiso V, Campanile F, Parisi G, Mariani B, Petrosillo N, Stefani S.** 2016. In vivo development of daptomycin resistance in vancomycin-susceptible methicillin-resistant Staphylococcus aureus severe infections previously treated with glycopeptides. Eur J Clin Microbiol Infect Dis **35:**625-631.

30. **Lin YT, Tsai JC, Yamamoto T, Chen HJ, Hung WC, Hsueh PR, Teng LJ.** 2016. Emergence of a small colony variant of vancomycin-intermediate Staphylococcus aureus in a patient with septic arthritis during long-term treatment with daptomycin. J Antimicrob Chemother **71:**1807-1814.

31. **Mishra NN, Yang SJ, Sawa A, Rubio A, Nast CC, Yeaman MR, Bayer AS.** 2009. Analysis of cell membrane characteristics of in vitro-selected daptomycin-resistant strains of methicillin-resistant Staphylococcus aureus. Antimicrob Agents Chemother **53:**2312-2318.

32. **Yamaguchi T, Suzuki S, Okamura S, Miura Y, Tsukimori A, Nakamura I, Ito N, Masuya A, Shiina T, Matsumoto T.** 2015. Evolution and single-nucleotide polymorphisms in methicillin-resistant Staphylococcus aureus strains with reduced susceptibility to vancomycin and daptomycin, based on determination of the complete genome. Antimicrob Agents Chemother **59:**3585-3587.

33. **Julian K, Kosowska-Shick K, Whitener C, Roos M, Labischinski H, Rubio A, Parent L, Ednie L, Koeth L, Bogdanovich T, Appelbaum PC.** 2007. Characterization of a daptomycin-nonsusceptible vancomycin-intermediate Staphylococcus aureus strain in a patient with endocarditis. Antimicrob Agents Chemother **51:**3445-3448.

34. **Iwata Y, Satou K, Tsuzuku H, Furuichi K, Senda Y, Sakai-Takemori Y, Wada T, Fujita S, Miyake T, Yasuda H, Sakai N, Kitajima S, Toyama T, Shinozaki Y, Sagara A, Miyagawa T, Hara A, Shimizu M, Kamikawa Y, Kaneko S, Wada T.** 2017. Down-regulation of the two-component system and cell-wall biosynthesis-related genes was associated with the reversion to daptomycin susceptibility in daptomycin non-susceptible methicillin-resistant Staphylococcus aureus. Eur J Clin Microbiol Infect Dis doi:10.1007/s10096-017-2999-3.
