# Supplementary material for: Gain-of-Function Mutations in the Phospholipid Flippase MprF Confer Specific Daptomycin Resistance
Source: mBio. 2018 Dec 18;9(6):e01659-18. doi: 10.1128/mBio.01659-18 (PMC6299216; doi:10.1128/mBio.01659-18)
Supplement: TABLE S3 [file mbo006184218st3.docx]

**Table S3 – Plasmids used in this study**

| **Plasmid** | **Characteristics** | **Short name in figure** |
| --- | --- | --- |
| pRB | *E. coli/S. aureus* shuttle vector pRB474 ([Bruckner, 1992](#_ENREF_8)) | nd |
| pRBmprF | *mprF* cloned in *E. coli/S. aureus* shuttle vector pRB474 ([Bruckner, 1992](#_ENREF_8)) | nd |
| pRB-S295L | Daptomycin resistance associated point mutations introduced in *mprF* by site directed mutagenesis and cloned in E. coli/S. aureus shuttle vector pRB474 ([Bruckner, 1992](#_ENREF_8)) | nd |
| pRB-P314L |  |  |
| pRB-S337L |  |  |
| pRB-T345A |  |  |
| pRB-V351E |  |  |
| pRB-I420N |  |  |
| pRB-L826F |  |  |
| pRB-D71A-T345A | T345A and additional flippase loss-of-function point mutations introduced in *mprF* by site directed mutagenesis and cloned in E. coli/S. aureus shuttle vector pRB474 ([Bruckner, 1992](#_ENREF_8)) | nd |
| pRB-R112A-T345A |  |  |
| pRB-E206A-T345A |  |  |
| pKT25-mprF | *mprF* gene encoding full length MprF C-terminally fused to adenylate cyclase fragment T25 in low copy vector pKT25 (Euromedex) ([Ernst et al., 2015](#_ENREF_10)) | MprF (Fig. 6) |
| pKT25-T345A | *mprF-T345A* gene encoding full length MprF-T345A C-terminally fused to adenylate cyclase fragment T25 in low copy vector pKT25 (Euromedex) | MprF-T345A (Fig. 6) |
| pKT25-flip | Truncated *mprF* gene encoding amino acids 1-320 of MprF (Flippase) C-terminally fused to adenylate cyclase fragment T25 in low copy vector pKT25 (Euromedex) ([Ernst et al., 2015](#_ENREF_10)) | Flip (Fig. 6) |
| pKT25-flip+2 | Truncated *mprF* gene encoding amino acids 1-393 of MprF (Flippase plus 2 TMS of synthase) C-terminally fused to adenylate cyclase fragment T25 in low copy vector pKT25 (Euromedex) ([Ernst et al., 2015](#_ENREF_10)) | Flip+2 (Fig. 6) |
| pKT25-flip+2-T345A | Truncated *mprF-T345A* gene encoding amino acids 1-393 of MprF (Flippase-T345A plus 2 TMS of synthase) C-terminally fused to adenylate cyclase fragment T25 in low copy vector pKT25 (Euromedex) | Flip+2-T345A (Fig. 6) |
| pUT18-mprF | *mprF* gene encoding full length MprF N-terminally fused to adenylate cyclase fragment T18 in high copy vector pUT18 (Euromedex) ([Ernst et al., 2015](#_ENREF_10)) | MprF (Fig. 6) |
| pUT18-T345A | *mprF-T345A* gene encoding MprF-T345A N-terminally fused to adenylate cyclase fragment T18 in high copy vector pUT18 (Euromedex) | MprF-T345A (Fig. 6) |
| pUT18-syn | Truncated *mprF* gene encoding amino acids 328-840 of MprF (Synthase) N-terminally fused to adenylate cyclase fragment T18 in high copy vector pUT18 (Euromedex) ([Ernst et al., 2015](#_ENREF_10)) | Syn (Fig. 6) |
| pUT18-syn-T345A | Truncated *mprF-T345A* gene encoding amino acids 328-840 of MprF-T345A (Synthase) N-terminally fused to adenylate cyclase fragment T18 in high copy vector pUT18 (Euromedex) | Syn-T345A (Fig. 6) |

**Bruckner R.** 1992. A series of shuttle vectors for Bacillus subtilis and Escherichia coli. Gene **122:**187-192.

**Ernst CM, Kuhn S, Slavetinsky CJ, Krismer B, Heilbronner S, Gekeler C, Kraus D, Wagner S, Peschel A.**2015. The lipid-modifying multiple peptide resistance factor is an oligomer consisting of distinct interacting synthase and flippase subunits. MBio **6**.
