# Supplementary material for: Gain-of-Function Mutations in the Phospholipid Flippase MprF Confer Specific Daptomycin Resistance
Source: mBio. 2018 Dec 18;9(6):e01659-18. doi: 10.1128/mBio.01659-18 (PMC6299216; doi:10.1128/mBio.01659-18)
Supplement: TABLE S4 [file mbo006184218st4.docx]

**Table S4 – Primers used in the study**

| Name | 5´ ⭢ 3´ sequence | Usage |
| --- | --- | --- |
| D71Afw | GTTATTCTATCAATGTATGCTGTGATTTTATCTAGAGCT | Forward primer for construction of pRB-D71A-T345A by site directed mutagenesis |
| D71Arev | AGCTCTAGATAAAATCACAGCATACATTGATAGAATAAC | Reverse primer for construction of pRB-D71A-T345A by site directed mutagenesis |
| R112Afw | CAGGCGTTGCAGCAATGGTTTATAAAAACTATACGC | Forward primer for construction of pRB-R112A-T345A by site directed mutagenesis |
| R112Arev | GCGTATAGTTTTTATAAACCATTGCTGCAACGCCTG | Reverse primer for construction of pRB-R112A-T345A by site directed mutagenesis |
| E206Afw | ACTTTAGTGTCGTGTGTTGCATGGTTAGCAGCTGCAGTT | Forward primer for construction of pRB-E206A-T345A by site directed mutagenesis |
| E206Arev | AACTGCAGCTGCTAACCATGCAACACACGACACTAAAGT | Reverse primer for construction of pRB-E206A-T345A by site directed mutagenesis |
| S295Lfw | GTAATTATTGCATTAATTTTATCATTATTTGAATTTGGTACATCAGCTAAG | Forward primer for construction of pRB-S295L by site directed mutagenesis |
| S295Lrev | CTTAGCTGATGTACCAAATTCAAATAATGATAAAATTAATGCAATAATTAC | Reverse primer for construction of pRB-S295L by site directed mutagenesis |
| P314Lfw | GGGATCTAAATACTTTATTCTTGCTAAAGATGTTACG | Forward primer for construction of pRB-P314L by site directed mutagenesis |
| P314Lrev | CGTAACATCTTTAGCAAGAATAAAGTATTTAGATCCC | Reverse primer for construction of pRB-P314L by site directed mutagenesis |
| S337Lfw | AAAATTCCATCATTATTATTAGCAATTTTAGTA | Forward primer for construction of pRB-S337L by site directed mutagenesis |
| S337Lrev | TACTAAAATTGCTAATAATAATGATGGAATTTT | Reverse primer for construction of pRB-S337L by site directed mutagenesis |
| T345Afw | GCAATTTTAGTATTCTTTGCAAGTATGATCTTTTTT | Forward primer for construction of pRB-T345A , pKT25-T345A, pKT25-flip+2-T345A, pUT18-T345A, pUT18-syn-T345A by site directed mutagenesis |
| T345Arev | AAAAAAGATCATACTTGCAAAGAATACTAAAATTGC | Reverse primer for construction of pRB-T345A, pKT25-T345A, pKT25-flip+2-T345A, pUT18-T345A, pUT18-syn-T345A by site directed mutagenesis |
| V351Efw | AGTATGATCTTTTTTGAAAATAACTTAACGATT | Forward primer for construction of pRB-V351E by site directed mutagenesis |
| V351Erev | AATCGTTAAGTTATTTTCAAAAAAGATCATACT | Reverse primer for construction of pRB-V351E by site directed mutagenesis |
| I420Nfw | TTCTTCACTTACGCTTCATATAATTTAATAACATGGTTAGCTATT | Forward primer for construction of pRB-I420N by site directed mutagenesis |
| I420Nrev | AATAGCTAACCATGTTATTAAATTATATGAAGCGTAAGTGAAGAA | Reverse primer for construction of pRB-I420N by site directed mutagenesis |
| L826Ffw | ATCGTAAAGATAATTCGTTCTGGGAATCACTTTCTAAAG | Forward primer for construction of pRB-L826F by site directed mutagenesis |
| L826Frev | CTTTAGAAAGTGATTCCCAGAACGAATTATCTTTACGAT | Reverse primer for construction of pRB-L826F by site directed mutagenesis |
